# Investigation of the Antibacterial, Anti-Biofilm, and Antioxidative Effect of *Piper betle* Leaf Extract against *Bacillus gaemokensis* MW067143 Isolated from Dental Caries, an In Vitro-In Silico Approach

**DOI:** 10.3390/microorganisms10122485

**Published:** 2022-12-15

**Authors:** Varda Jalil, Maryam Khan, Syed Zeeshan Haider, Saba Shamim

**Affiliations:** Institute of Molecular Biology and Biotechnology, The University of Lahore, Defence Road Campus, Off-Bhobatian Chowk, Lahore 54000, Pakistan

**Keywords:** *Piper betle*, dental caries, *Bacillus*, antibacterial activity, scanning electron microscopy, molecular docking

## Abstract

Among oral diseases, dental caries is one of the most frequent to affect human health. The current research work aimed to ascertain the antibacterial, anti-biofilm, and antioxidative potential of *Piper betle* leaf extract against bacteria isolated from dental caries. Analysis for the presence of phytochemical compounds revealed compounds, such as tannins, steroids, phenolic compounds, and alkaloids, which were also confirmed by TLC and FTIR. GC-MS analysis elucidated the presence of 20 phytocompounds, among which were some well-reported bioactive compounds. The chloroform extract of *P. betle* demonstrated good antibacterial activity (7 mm) and minimum inhibitory concentration (MIC) (100 mg mL^−1^) against *Bacillus gaemokensis* MW067143, which was the frequent biofilm producer among isolated bacterial strains. Fractions of the extract were isolated through column chromatography, after which the antibacterial activity was again evaluated. Spirost-8-en-11-one,3-hydroxy(3β,5α,14β,20β,22β,25R), an oxosteroid in nature, was observed to exhibit remarkable antibacterial potential (12 mm) against *B. gaemokensis*. Bacterial cells treated with *P. betle* extract had elevated SOD, APOX, POX, and GR activity, while its proteolytic activity against whole bacterial proteins was pronounced with the suppression of several proteins (50, 40, 15, and 10 kDa) in SDS-PAGE. Bacterial cells treated with *P. betle* extract demonstrated decreased growth, while the extract was also observed to exhibit inhibition of biofilm formation (70.11%) and demolition of established *B. gaemokensis* biofilms (57.98%). SEM analysis revealed significant changes to bacterial morphology post treatment with *P. betle*, with cellular disintegration being prominent. In silico network pharmacology analysis elucidated proteins like ESR1 and IL6 to be majorly involved in biological pathways of dental caries, which also interact with the protective ability of *P. betle*. Gene Ontology (GO) terms and KEGG pathways were also screened using enrichment analysis. Molecular docking demonstrated the highest binding affinity of Spirost-8-en-11-one,3-hydroxy-,(3β,5α,14β,20β,22β,25R) with bacterial proteins FabI (−12 kcal/mol), MurB (−17.1 kcal/mol), and FtsZ (−14.9 kcal/mol). Therefore, it is suggested that *P. betle* can serve a potentially therapeutic role and could be used in the preparation of herbal formulations for managing bacterial flora.

## 1. Introduction

The existence of teeth in the oral cavity presents itself to be one of the most distinctive features of body anatomy. Saliva, mucosal, soft and hard tissue linings of teeth, and the diverse microbial communities are just some of the ecological niches that pertain to the oral cavity [1]. The protrusion of teeth from the mucosal tissue in the oral cavity provides a solid surface for the formation of bacterial biofilms [2]. Oral biofilms are formed by the complex network of interspecific competition between these microbial communities [3] embedded in extracellular polymeric substances (EPS) matrices comprised of biological molecules, such as carbohydrates, proteins, and nucleic acids, which act as scaffolds to the structure [4]. The microflora of the human body usually comprises saprophytic microorganisms, which start to colonize the human body even almost immediately after the birth of an individual, where bacteria are the dominant residents. They have an astonishing capability to attach and inhabit epithelial cells and to reproduce, and they are found readily in the human body. The oral microflora is home to more than 700 species of bacteria, as well as fungi, viruses, protozoa, and archaea [5]. While bacterial taxa like Bacteroidetes and Firmicutes usually comprise the salivary microbiota, many other bacterial species are home to the oral cavity, which usually synergistically contribute with fungi to the formation of dental plaque and biofilms [6,7].

Dental caries is one of the most prevalent type of noncommunicable diseases, with more than 3.5 billion people affected worldwide [8,9]. Though significant improvements have been observed in regard to oral health and awareness, a high burden of this disease still subsists [10]. It is a chronic disease marked by demineralization of dental surfaces, which are the result of acidic niches produced by metabolic products of biofilms [11]. It can affect any gender and age and is notorious for causing pain, discomfort, and impacting routine life like other oral cavity-related diseases [12]. Caries is a multi-stage disease that manifests over time with various agents of causation, such as dietary intake, lifestyle, smoking, socioeconomic status, and poor oral hygiene, all contributing significantly to its widespread distribution [13]. Apart from these factors, the presence of bacterial species, carbohydrates, and vulnerable tooth surfaces are actively involved in the progression of caries [14,15]. Established treatments for dental caries include the use of fluorides and control of plaque through professional dental treatments and mouthwashes. However, the majority of affected people, especially from developing or third-world countries, lack sufficient awareness and dexterity to follow on these practices. Furthermore, agents like chlorhexidine gluconate in mouthwashes exhibit several undesirable side effects, such as elevated mineral uptake, irritation of mucosal surfaces, staining of teeth, and alteration of taste [16].

Alternatively, using medicinal plants and their bioactive compounds for the effective management and prevention of oral diseases like dental caries has been investigated over the years, which implies that they have therapeutic applications and minimal side effects. While the earliest known use of herbal therapy to treat oral diseases is reported to date back to traditional Indian and Chinese medicine [17], modern drugs comprising of single compound or a combination with others have proven plants and herbs to be effective against dental caries [18]. *Piper betle* L. is a perennial native plant to Asian and Southeast Asian countries [19]. It has characteristic heart-shaped leaves and is a member of the Piperaceae family, which houses more than a thousand plant species routinely found and grown in countries like India, Sri Lanka, and Bangladesh [20]. Moreover, it has been used as a medicinal plant as its therapeutic potential has been reported in many studies conducted over the years. Betel leaves, due to their aroma, are routinely used in the treatment of bad breath and toothache. They are also used for the treatment of various medical conditions like conjunctivitis, itches, boils, abrasion, cuts, and wounds, as well as being used as a homeopathic medicine for treating female infertility [21]. Along with its established antimicrobial activity, it also exhibits gastro- and hepato-protective activities, respectively [22]. Betel leaves have significant value in the pharmaceutical industry as they are reported to possess aromatic, digestive, expectorant, euphoria-inducing, stimulative, antibacterial, antiprotozoal, carminative, aphrodisiac, and antifungal properties [23].

This study aimed to evaluate the antibacterial, anti-biofilm, and antioxidative effect of *P. betle* extract against bacteria isolated from dental caries. Furthermore, phytochemical characterization and in silico studies were performed to elucidate the types of phytochemical compounds present in *P. betle* extract and to decipher the mechanism of action and binding affinity of these compounds with selected target proteins. The abbreviations used in this manuscript are given in Table 1.

## 2. Results

### 2.1. Isolation, Purification, and Characterization of Bacterial Flora from Caries Samples

**Figure 1:**
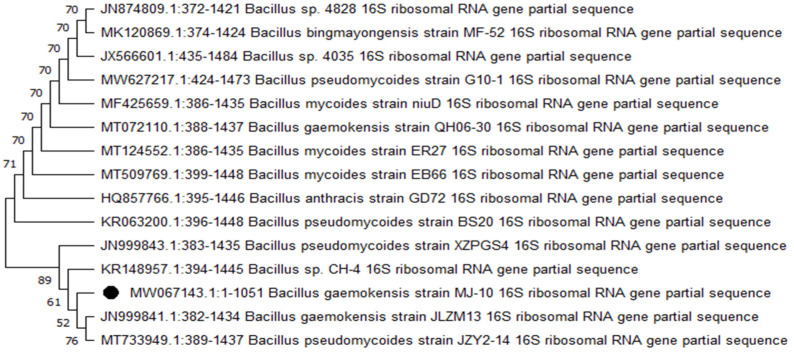
Maximum-likelihood tree of *B. gaemokensis* using neighbor-joining method. Bootstrap values are expressed as a frequency of 1000 replicates, and values less than 50% are not shown.

For this study, a total of 2700 samples (saliva and swab) were collected among which 2500 were from caries patients, while 200 were control patients. Among these 2500 cariogenic samples, only 1900 were positive for the bacterial growth (Table 2). From these 1900 positive cultures, 15 different bacterial species were isolated and characterized at cultural, morphological, biochemical, and molecular levels. Two bacterial species were found in control samples. The details are given in Table 3. From these 15 bacterial species, only one bacterial isolate was found as the most frequent, producing the characteristic thick biofilm. The isolate was identified as *Bacillus gaemokensis* after subsequent biochemical and molecular characterization (Figure 1). The 16s rRNA sequence was submitted to NCBI Genbank under the accession number MW067143. Figure 1 demonstrates the phylogenetic tree constructed through MEGA11 software, illustrating comparison and closest strain homology among *B. gaemokensis* and other members of *Bacillus* spp.

### 2.2. Phytochemical Screening of P. betle Extract

Chloroform extract of *P. betle* was subjected to various tests for the presence of various phytocompounds. The results demonstrated the presence of several phytocompounds, such as alkaloids, flavonoids, steroids, tannins, and phenolic compounds, as shown in Table 4.

### 2.3. TLC Analysis of P. betle Extract

**Figure 2:**
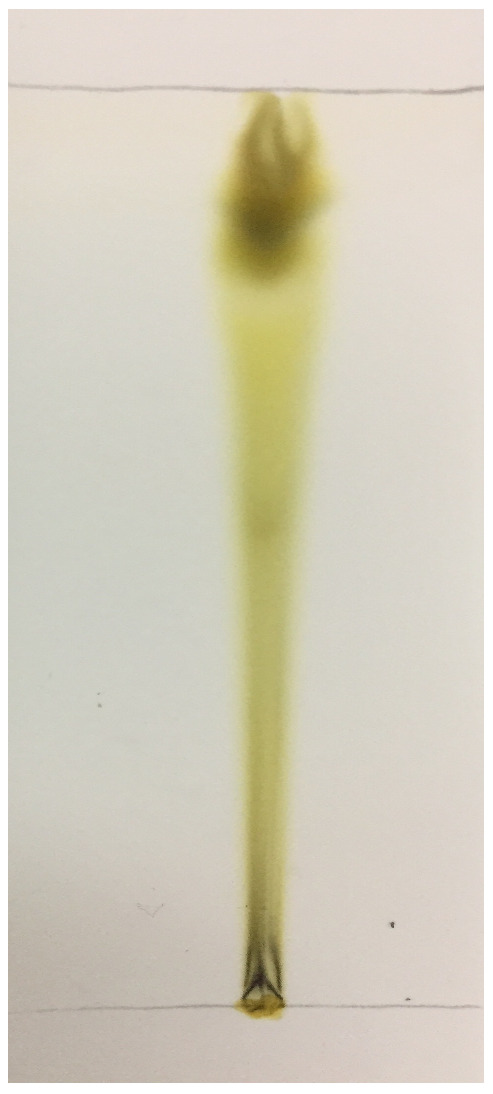
TLC analysis of *P. betle* chloroform extract.

Thin layer chromatography (TLC) yielded the presence of multiple compounds when they were visualized under UV light. The Rf values of the compounds found in the chloroform extract were 0.9, 0.81, and 0.62, respectively, as shown in Figure 2.

### 2.4. FTIR Analysis of P. betle Extract

**Figure 3:**
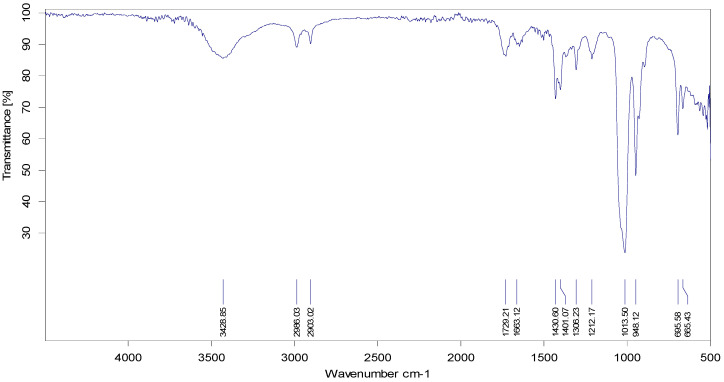
FTIR spectra of *P. betle* extract.

Functional groups of *P. betle* extract were characterized by FTIR analysis, and the major peaks of absorption (3428.85, 2986.03, 2903.02, 1729.21, 1663.12, 1430.60, 1401.07, 1306.23, 1212.17, 1013.51, 948.19, 695.58, and 665.43 cm^−1^) were identified and characterized according to the nature of the phytochemical compounds present in the extract (Figure 3).

### 2.5. GC-MS Analysis of P. betle Extract

GC-MS analysis yielded the presence of 20 compounds, the details of which are provided in Table 5.

### 2.6. Minimum Inhibitory Concentration (MIC)

Different concentrations were tested for the MIC value for *P. betle* extract. Bacterial growth was inhibited at the concentration of 100 mg mL^−1^, which was stated as the MIC value for *B. gaemokensis*. This concentration was thus chosen as the extract concentration for the rest of the study (Table 6).

### 2.7. Antibacterial Activity of P. betle Extract

The antibacterial potential of *P. betle* extracts against *B. gaemokensis* was observed through well-diffusion assay. The chloroform extract greatly eradicated the growth of the bacterium, giving a zone of inhibition of 7 mm, while no results were observed in the methanol extract. Ethanol extract also demonstrated inhibition of the bacterium, albeit in a lesser amount. For this study, chloroform extract was thus chosen for further experiments. DMSO did not yield any result while zones of inhibition were yielded by Piperacillin/Tazobactum (positive control) (Table 7).

### 2.8. Isolation and Purification of Bioactive Compound from P. betle Extract

**Figure 4:**
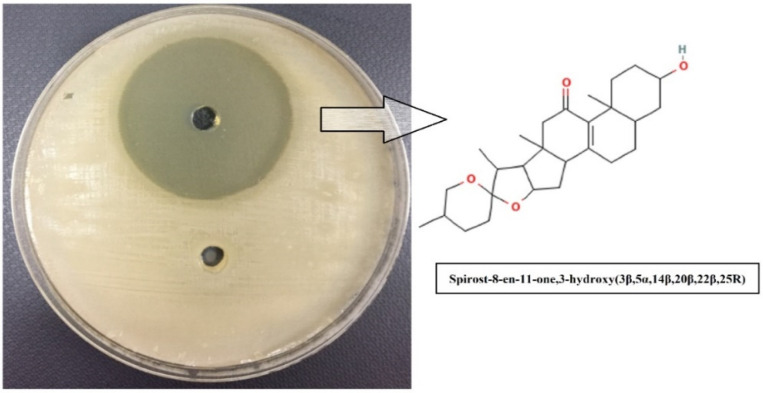
Zone of inhibition of isolated compound Spirost-8-en-11-one,3-hydroxy(3β,5α,14β,20β,22β,25R) (chemical structure shown) from *P. betle* extract against *B. gaemokensis*.

Since the chloroform extract was observed to give a zone of inhibition, the isolated fractions of the extract were tested for their antibacterial activity against *B. gaemokensis*. The isolated compound was characterized as Spirost-8-en-11-one,3-hydroxy-,(3β,5α,14β,20β,22β,25R), which effectively inhibited the growth of the bacterium (Figure 4, Table 8).

### 2.9. Estimation of Antioxidative Enzymes

**Figure 5:**
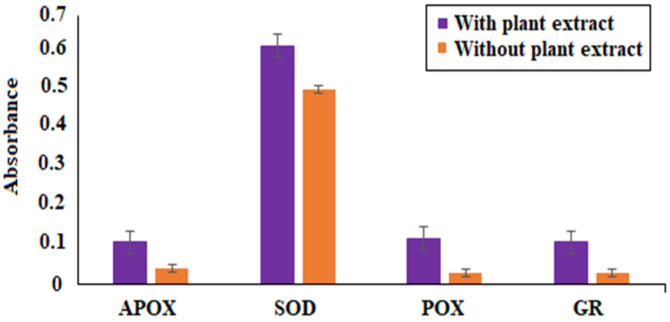
Expression of antioxidative enzymes of *B. gaemokensis* in the presence and absence of *P. betle* extract.

The quantification of antioxidative enzymes was carried out in the presence of *P. betle* chloroform extract. In *B. gaemokensis*, expression of SOD, APOX, GR, and POX enzymes was seen to be elevated when cells were treated with the extract, as shown in detail in Figure 5.

### 2.10. Time-Kill Assay

**Figure 6:**
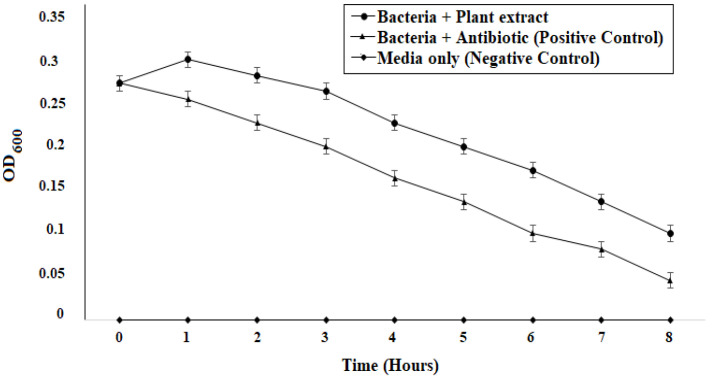
Time-kill assay of *B. gaemokensis* cells in the presence *P. betle* extract and controls.

A decreasing trend in the growth of *B. gaemokensis* cells was demonstrated with *P. betle* extract, which was found comparable with the antibiotic (positive control) (Figure 6), indicating that the extract effectively reduced the growth of the bacterium.

### 2.11. Inhibition and Demolition of B. gaemokensis Biofilm

**Figure 7:**
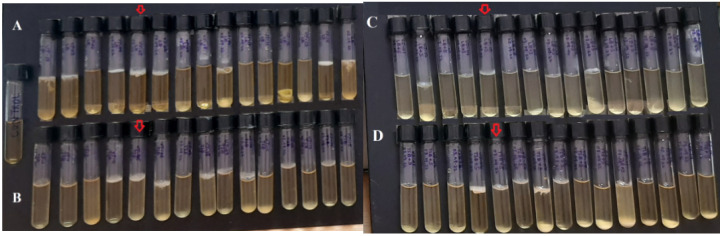
Bacterial strains inoculated in Luria Bertani (LB) broth (Row A), Tryptic Soy Broth (TSB) (Row B), LB + Sucrose (Row C), and LB + TSB (Row D). Control is shown in the tube at left side. Red arrows showing the *B. gaemokensis* growth in all four different growth media.

The biofilm formation by all 15 cariogenic strains is given in the Figure 7. 

**Figure 8:**
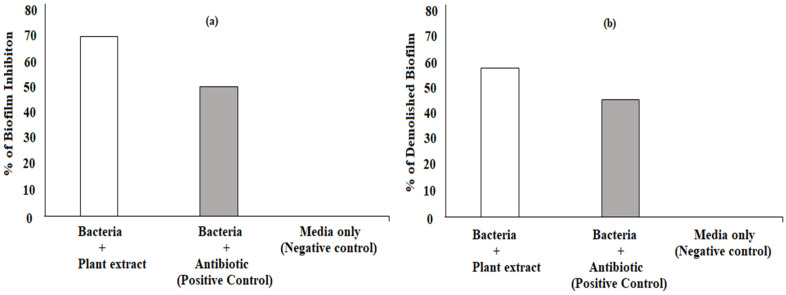
Biofilm assays of *B. gaemokensis* in the presence of *P. betle* extract and controls. (**a**) Inhibition of biofilm formation; (**b**) demolition of established biofilm.

*P. betle* chloroform extract was observed to exhibit more inhibitory action (70.11%) on the biofilm formation of *B. gaemokensis* as compared to the antibiotic used (50.45%) (Figure 8a). In a similar fashion, *P. betle* extract, upon addition, was responsible for causing the demolition of established *B. gaemokensis* biofilm (57.98%), as shown in Figure 8b.

**Figure 9:**
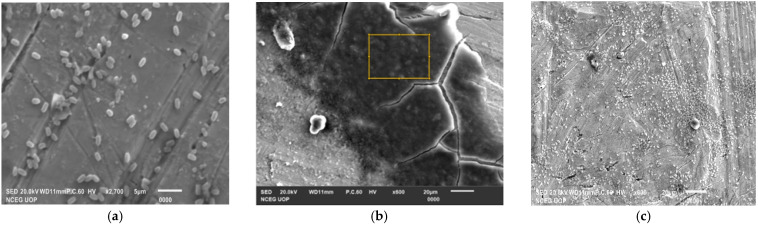
SEM analysis of (**a**) 24 h *B. gaemokensis* culture, (**b**) after 48 h, and (**c**) a close view of the same 48 h old culture.

SEM analysis of 24 h and 48 h old cultures of *B. gaemokensis* is shown in Figure 9a–c.

### 2.12. Protein Estimation

**Figure 10:**
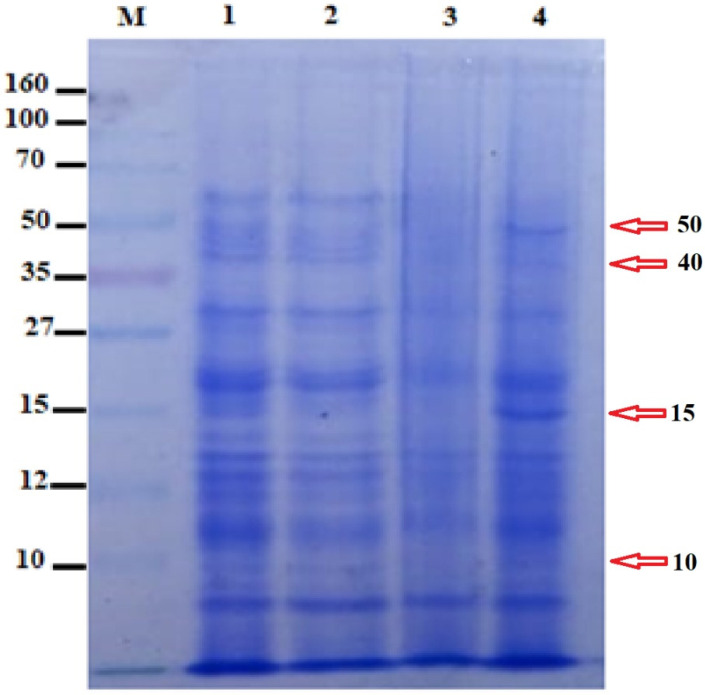
SDS-PAGE analysis of *P. betle* extracts (M- marker, Lane 1-ethanol extract, Lane 2-methanol extract, Lane 3-chloroform extract, Lane 4-control).

SDS-PAGE revealed the expression of several proteins in the ethanol, methanol, and chloroform extracts of *P. betle*, respectively, and the control sample. The chloroform extract was observed to suppress the expression of various proteins (50, 40, 15, and 10 kDa) in a more pronounced way as compared to other extracts and control sample, as shown in Figure 10. The band of 15 kDa was observed in the control sample, which was not found to be expressed in other samples containing bacteria and the extracts of *P. betle*.

### 2.13. Scanning Electron Microscopy

**Figure 11:**
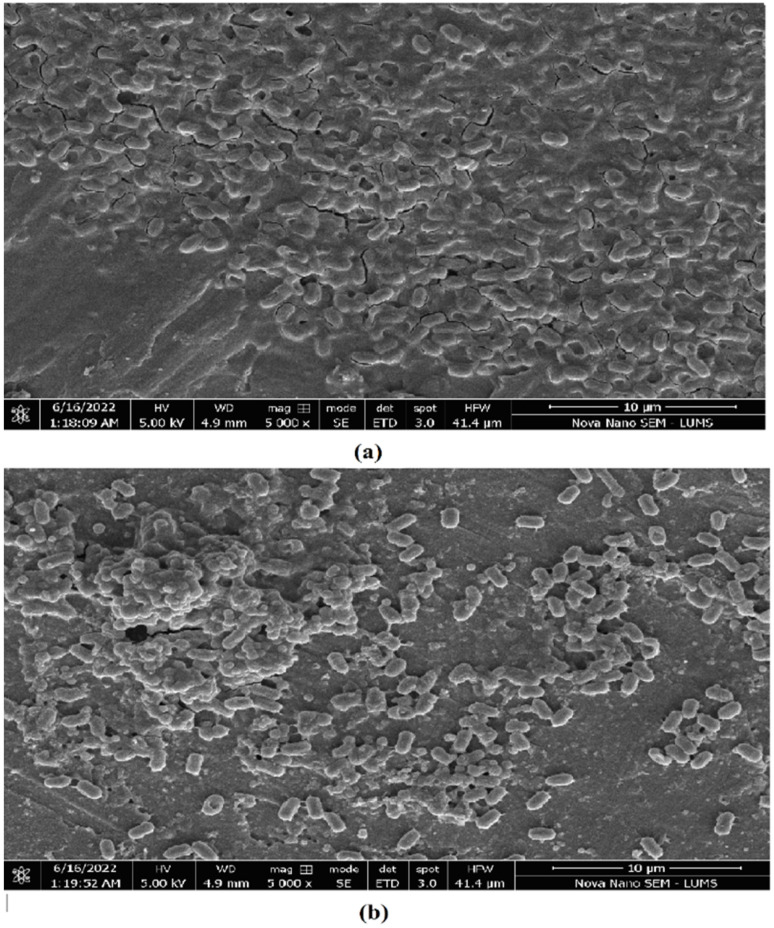
SEM micrographs of *B. gaemokensis* cells in the (**a**) absence and (**b**) presence of *P. betle* chloroform extract.

The difference in the morphology was observed in both SEM micrographs obtained for control and treated bacterial cells (Figure 11a,b). *B. gaemokensis* cells were treated with *P. betle* chloroform extract (100 mg mL^−1^) for 24 h, after which the reduction of the cell number is clearly shown in Figure 11b, depicting the antibacterial activity of *P. betle*.

### 2.14. In Silico Studies

#### 2.14.1. Network Pharmacology

**Figure 12:**
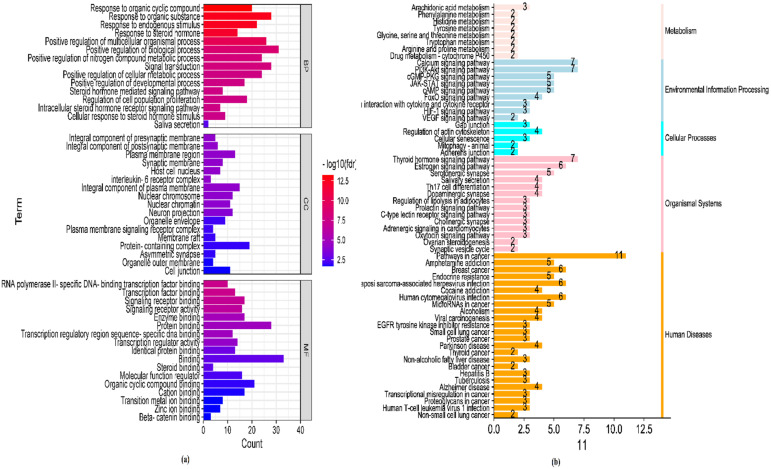
Enrichment analysis of (**a**) Gene Ontology (GO) terms and (**b**) KEGG pathways of *P. betle* extract for dental caries.

The PPI network was constructed using STRING database, with a medium network probability score of 0.400, 35 nodes, and 157 edges. The node degrees of the target proteins are provided in Table 9. The average node degree was observed to be 8.97. The *p*-value of the enrichment network was found to be negligibly small. Target proteins, such as Sarcoma (Schmidt-Ruppin A-2) viral homolog (SRC), Estrogen receptor 1 (ESR1), Progesterone receptor (PGR), and Interleukin 6 (IL6), had high node degree values of 18, 13, and 12, respectively. Gene Ontology (GO) term and KEGG analysis elucidated 399 (biological processes), 67 (molecular functions), and 39 (cellular components) significantly enriched GO terms (Figure 12a), while KEGG pathway analysis revealed 61 significantly enriched pathways, the details of which are shown in Figure 12b.

#### 2.14.2. Molecular Docking

The target sites of *B. gaemokensis* were searched and extensively screened, after which Enoyl-acyl carrier protein reductase (FabI) (PDB ID: 4FS3), Mur B (UDP-N- Acetylenolpyruvylglucosamine Reductase) (PDB ID: 1HSK), and FtsZ (PDB ID: 6KVP) were selected. All three target sites are commonly found in both Gram-positive and -negative bacteria and are a part of the bacterial cytoplasm. In this study, Spirost-8-en-11-one,3-hydroxy(3β,5α,14β,20β,22β,25R), diisooctyl phthalate, and caryophyllene demonstrated the highest binding energies, as shown in Table 10.

## 3. Discussion

Oral cavity is the largest organ after the gut that inhabits more than 700 microorganisms [24] including bacteria, fungi, etc. [5,25]. Some of these organisms actively form biofilms on various oral surfaces such as dental prostheses and epithelial cells [26]. They are also found to be the causative agents of oral diseases including tooth decay (caries), gum-related infections (gingivitis and periodontitis), and root canal infections (endodontitis). These diseases, which are most commonly occurring oral diseases in humans of all ages [27], comprise a serious global health issue [28]. Demineralization of tooth enamel is caused by the elevated acid production during glycolysis after intaking high carbohydrate food. The tooth enamel is restored by remineralization, which occurs after alkalinization and ultimately leads to the diffusion of acids from biofilms. These acids are buffered by salivary bicarbonate, salivary peptides, and the bacterial metabolism of urea and arginine. The stage comes where the acidification outweighs the alkalinization, which leads to the dental caries. Ultimately, the pH values lower and thus prolonged dental caries persists [29]. The NIH recommends brushing, flossing, and mouthwash usage on the regular basis in order to avoid the oral cavity diseases [30].

In this study, total 2500 cariogenic samples were collected, among which 1900 were positive cultures, while 600 were negative cultures (Table 2). In the 165 positive culture samples, a different frequency of the various bacterial species was observed. Their molecular characterization and prevalence are given in the Table 3. According to this study, the highest frequency of *S. mutans* was observed, followed by *S. sobrinus*, *S. aureus*, *B. gaemokensis*, *B. cereus*, *B. subtilis*, *S. haemolyticus*, and *B. flexus,* and the lowest frequency was *L. salivarius*, *L. rhamnosus,* and *P. stutzeri* (Table 3). *S. aureus,* as a resident of the oral cavity of healthy adults, was already reported [31]. The role of *Bacillus acidophilus* as a cariogenic agent was reported earlier this century [32,33]. Early studies reported diverse microorganisms as cariogenic agents like *Streptococcus mutans* [34,35,36], *S. sanguinis*, *Bacillus cereus* [37], *L. acidophilus* [38], *S. aureus* [39,40,41,42], *S. sobrinus* [43,44], *Pseudomonas stutzeri* [45], *S. haemolyticus* [46]. *L. rhamnosus,* and *L. salivarus* found in the current study, which belongs to the casei group of *Lactobacillus* [47]. The biofilms formed by *B. cereus* were reported by Majed et al. [48].

*B. subtilis* from the oral cavity was reported previously [38,49,50,51]. Biofilm formation by oral cavity-inhabited *B. subtilis* was reported by Jain et al. [52]. According to Shaw [53], *Bacillus fusiformis* was one of the causative agents of acute necrotizing ulcerative gingivitis, leading to infection [54]. *B. licheniformis* as a dental cariogenic agent was reported by Rostinawati et al. [55]. *B. acidophilus* was reported as cariogenic agent by Tucker [56]. Biofilm formation by *Bacillus* species was reported previously [57]. *Bacillus subtilis* from the oral cavity was reported by Yamane et al. [58]. Biofilm of dental *B. subtilis* [52] and *B. licheniformis* [55] was reported. The role of *Bacillus* in dental caries or *Bacillus* as a cariogenic agent can be hypothesized from its adherence to the dental enamel, followed by its colonization, which ultimately results in the form of film or biofilm containing a large number of *Bacillus* cells. Their source of energy is the remaining of the food particles that are left behind after taking meals. Out of these 15 bacterial species isolated in this study, *Bacillus gaemokensis* was selected on the basis of its thick biofilm feature. In simple words, first the bacterial species were isolated and characterized at the molecular level; then, their biofilm forming ability was observed. Biofilm formation was the selective criteria for the selection of bacteria for further studies (Figure 7). Here, *B. gaemokensis* was selected on this basis. *B. gaemokensis* is environmental microorganism. It was first reported by Jung et al. [59] from tidal flat sediment of the Yellow Sea. Similarly, *Bacillus pumilis* and *Bacillus flexus* are the environmental microorganisms, among which the latter is known for its biofilm production [57].

In our study, the most potent biofilm producer among the bacterial isolates was identified to be *Bacillus gaemokensis*, which was also found to be the fourth most frequent isolate among collected samples (Table 3). The use of medicinal plants for treating dental caries and other diseases has been well reported over the years, mediated by the action of various bioactive compounds reported in the composition of these plants [58]. The antibacterial activity of *Piper betle* and its extracts has been reported against various pathogens [60,61,62,63]. In this context, we investigated the antibacterial and anti-biofilm potential of *Piper betle* extract against *B. gaemokensis* isolated from dental caries. Nature has gifted plants with the presence of different substances that aid in the biological activities of living beings. These substances, known as phytochemical compounds, are largely responsible for the close association between their bioactivity and their therapeutic potential. These substances hold no nutritional value but are equipped with potent antimicrobial and other properties, which enable them to ward off disease [64]. In this study, *P. betle* extract was demonstrated to contain various phytochemical compounds (Table 4). TLC analysis elucidated the presence of three compounds with different Rf values (Figure 2). Aara et al. [65] reported the Rf value of eugenol to be 0.84, which is almost equivalent to the Rf value observed in our study, thereby confirming the presence of eugenol. FTIR analysis demonstrated the absorbance peak at 3428.25 cm^−1^, which corresponds to the –OH group found in phenolic compounds (Figure 3). Additional bands of 2986.03 and 2903.03 cm^−1^ are due to the C-O-H bonds. The rest of the bands (up to 1212.17 cm^−1^) mark the presence of aromatic compounds, respectively. The band at 1013.50 cm^−1^ is the sharpest, which can be associated with C-O stretching. Singh et al. [66] also confirmed the presence of compounds like alcohols, phenolic compounds, alkanes, and alkenes, which was similar to our study. GC-MS analysis elucidated the presence of 20 phytocompounds, the majority of which are widely reported bioactive compounds having known antibacterial potential (Table 5). The isolated fractions were investigated for their antibacterial activities against *B. gaemokensis*, of which Spirost-8-en-11-one,3-hydroxy(3β,5α,14β,20β,22β,25R) was observed to demonstrate the most effective antibacterial action against the bacterium (Figure 4). It has been suggested that the formation of the distinct structure of *B. subtilis* biofilms relies on the ability of the bacterial cells to heterogeneously differentiate into motile, ECM-producing, and spore-forming cells, all within the bacterial colonies. This ability and the eventual phenotype are fundamentally pre-determined by various factors, such as temperature, availability of nutrients, oxygen, growth media, and availability of sugars, along with other factors [67,68]. The attachment of bacterial cells to the tooth surface leads to the formation of film or biofilm, which is composed of the dietary particles as well. Among various dietary constituents, sucrose is considered to be the most cariogenic in nature owing to its fermentable nature, resulting in low pH of the dental premises [69]. The shift in resident microflora to more cariogenic one is in accordance with ecological plaque hypothesis [70]. It ultimately leads to dental demineralization. Dental biofilms are directly affected by the dietary fermentable sugars including glucose, sucrose, maltose, fructose, etc. However, the clear direct effect of sucrose on physiology and biochemistry of the biofilm formation leading to enhancing dental caries has already been reported [69]. Sucrose is a cariogenic dietary carbohydrate [71]. Its metabolism leads to the acid production, which results in an acidic environment, thus promoting cariogenic aciduric bacterial flora and not alkali-producing bacteria, which causes dental demineralization due to formation of biofilms by aciduric bacterial species as observed in this study (Table 3, Figure 7) when the growth media (LB and TSB) were supplemented with sucrose.

The MIC of the extract was reported to be 100 mg mL^−1^, which was then selected as the concentration to conduct further experiments (Table 6). Antibacterial activity of *P. betle* extracts (ethanol, methanol, and chloroform) demonstrated effective zones of inhibition, with the most effective activity being of the chloroform extract, which was then chosen for further analysis (Table 7). In a previous research work, *P. betle* extract was examined against dental plaque bacteria, which demonstrated its bacteriostatic effect against frequent oral pathogens [72]. Rahman et al. [73] also reported *P. betle* leaf extract to be effective against *B. subtilis* and *Staphylococcus aureus*, respectively. The inhibitory action of *P. betle* extract was observed on *B. gaemokensis* and its established biofilm (Figure 8a,b). In the biofilm experiments, the SEM images of 24 and 48 h old culture of *B. gaemokensis* is shown in Figure 9(a–c). The protective effects of *P. betle* in the oral cavity have rendered the plant to be effective in the prevention of biofilm formation and reduction of gingival inflammation [74,75]. The time-kill assay also marked a trend of decrease in bacterial growth when *P. betle* extract was added, which was also observed in other similar research findings [76,77] (Figure 6). Various antioxidants like SOD, CAT, and GPx prevent, repair, and regulate the detrimental effects of oxidative stress by acting on their radical scavenging activity [78]. Therefore, the role of antioxidants in the regulation of oxidative stress holds promise from a therapeutic standpoint [79]. The expression of enzymes like APOX, POX, SOD, and GR in *B. gaemokensis* were observed to be remarkably induced when bacterial cells were treated with *P. betle* extract (Figure 5). SOD activity was the most pronounced, while APOX, POX, and GR activity were roughly the same. The findings of our study agreed with Abrahim et al. [80], where the increased expression of SOD was reported in the presence of *P. betle* leaf extract. The elevated expression of the SOD enzyme demonstrates their ability to remove or scavenge superoxide anions, leading to the alleviation of reactive oxygen species (ROS). Profiling of bacterial proteins gives an insight into their complex genome, due to which the quantification and evaluating the expression of whole protein (treated and/or untreated) also serves a significant role when performing comparative analyses [81]. In our study, the suppression of bacterial proteins in extract-treated cells suggests the proteolytic activity of the extract (Figure 10). This activity can be attributed to the degradation of proteins by antibacterial (bioactive) compounds [82,83]. Moreover, it is important to note that the disappearance of whole cell proteins in treated bacterial cells indicates that their synthesis is not affected, but rather there is an inhibition of protective enzymes that sustain the cellular integrity of the bacterium [84]. SEM analysis of treated and untreated *B. gaemokensis* cells with *P. betle* extract suggests noticeable morphological changes to the structure of bacterial cells (Figure 11). Untreated cells were observed to be smooth and intact, while treatment with *P. betle* extract rendered distortion in the cell structure, whereby cells became swollen and disintegrated. Formation of pores was apparent, as well as cell lysis. Cellular debris in the surroundings of the cells were also visible after treatment. Ramasamy et al. [85] also reported the disintegration of cellular structure after treatment with plant extract.

The mechanism of action of *P. betle* extract against *B. gaemokensis* was ascertained by in silico studies, while docking was used to predict the binding of phytocompounds with potential target sites found in the bacterium. A systematic study comprising network construction and its visualization aided in understanding the signaling pathways involved in the action of *P. betle* against dental caries. The PPIN revealed a diverse array of interacting moieties with *P. betle* and its primary bioactive compounds like eugenol, caryophyllene, and phytol. Mediators like ESR1, SRC, and IL6 demonstrated the strongest association to bioactive compounds, stating their role in the mechanism of action of *P. betle* (Table 9). Dental fluorosis is linked to human ESR1 because estrogen or its receptors affect the activity of ameloblasts, which directly leads to the development of dental fluorosis. Similarly, GO terms and KEGG pathways are also crucial in understanding which pathways and genes are involved in the mechanism of action and up/downregulation of biological pathways involved with *P. betle* (Figure 12).

Molecular docking is a method that unravels the interactive abilities of a molecule with its target, which is customarily a protein. In this study, 20 compounds were selected for molecular docking, as revealed by the GC-MS analysis of *Piper betle* extract (Table 10). FabI is a well-reported protein that is involved in cell wall and cell membrane integration through the fatty acid biosynthesis pathway (FAS-II), which is attributable to the synthesis of lipids and fatty acids, the major primary constituents of the bacterial cell wall [86]. FtsZ serves its key role in the cytokinetic machinery of the bacterial cytoskeleton, via the formation of a “Z” ring located at the center of the cell, which functions to constrict the cell division of the cell [87]. In this study, Spirost-8-en-11-one,3-hydroxy(3β,5α,14β,20β,22β,25R) was observed to exhibit the highest binding energy against the three target sites. This in silico approach can be validated by previously mentioned results, whereby the compound was isolated and investigated for this antibacterial activity. Therefore, the docking analysis predicted that the compounds work with these proteins, which are involved in the essential regulation of the metabolism of bacteria, suggesting that targeting this mechanism may be one of the main routes that plant compounds use to exert their pharmacological and antimicrobial actions and pathological aspects on bacterial species and human beings. In vivo studies can help us to gain an insight into the practicability of Spirost-8-en-11-one,3-hydroxy(3β,5α,14β,20β,22β,25R) as an anticariogenic agent.

## 4. Materials and Methods

### 4.1. Sample Collection Criteria and Sampling Details

For patients with dental caries, the lingual and proximal surfaces of decaying teeth were scraped using a sterile metal excavator. Control samples were similarly acquired, but from healthy teeth. The samples were brought to the Microbiology Laboratory at the Institute of Molecular Biology and Biotechnology (IMBB) and were processed immediately [88]. Sanction for this work was approved from the Human Research Ethics Committee of the University of Lahore, Pakistan. The inclusive and exclusive criteria of the sampling are given below.

#### 4.1.1. Inclusive Criteria

Inclusion criteria include all healthy individuals of both genders (10–50 years) with dental caries.

#### 4.1.2. Exclusive Criteria

The immunosuppressive patients showing the history of any chronic morbid and debilitating diseases including diabetes, hypertension, liver problem, etc. were excluded from this study.

#### 4.1.3. Calculation of Dental Caries Scoring for the Selection of Patients

Keeping in mind the inclusive and exclusive criteria, the dental caries scoring was calculated. For this purpose, the patient was seated on dental chair, examined under bright light with mouth mirrors and gauze. Caries was identified and the caries index was expressed as DMFT score as recommended by WHO report on oral health survey [89]. The DMFT score calculation is given below:

Calculation of DMFT:

1- (Score for individual) DMF = D + M + F

    2- (Score for population) DMF(Mean) = Total DMFT

                             Total No. of the pts examined

Max. score: DMFT = 32

        Min. score: DMFT = Null or absent caries

#### 4.1.4. Collection of Samples

In total, 2500 samples (saliva and swab) were collected by a qualified dental surgeon. Additionally, 200 samples (control) were collected.

##### Saliva Samples

The patient was advised a day before for giving his/her sample before drinking and eating anything usually early in the morning. The saliva was collected in a sterilized falcon tube by spitting method. The saliva was homogenized by centrifugation at 3000× *g* for 15 min at room temperature. The aliquots of the clarified supernatants were stored at −80 °C.

##### Swab Method

An excavator was used for the collection of samples from the smooth surface and pit and fissure caries with great care as chances of contamination existed. A sterilized swab was rub on it 4–5 times followed by swabbing on the media plate.

### 4.2. Isolation and Characterization of Bacterial Flora from Caries Samples

Luria Bertani (LB) agar medium was used for isolating cariogenic bacteria from samples, which were swabbed on the agar surface. Bacterial colonies were observed for their distinct morphological characteristics (size, margin, elevation, texture, smell, shape, pigmentation) and were streaked repeatedly on LB agar until purified colonies were obtained. Gram staining was performed to determine the Gram morphology of the isolates. In the next step, the isolates were subjected to various biochemical tests (catalase, coagulase, urease, indole, citrate, motility, methyl red/Voges Proskauer, nitrate reduction) for their biochemical identification [88].

### 4.3. Molecular Characterization of Selected Bacteria 

The extraction of genomic DNA of selected isolates was performed using the CTAB method of Wilson [90]. The isolated genomic DNA samples were proceeded on agarose gel (1%) using horizontal gel electrophoresis according to the method of Sambrook and Russell [91]. Fluorescence under ultraviolet (UV) light marked the presence of gDNA when observed under a transilluminator. 16S rRNA sequencing of the samples was confirmed from Macrogen^®^, Seoul, Republic of Korea, after which the results were submitted to National Center for Biotechnology Information GenBank. The nucleotide sequences of 16s rRNA genes were evaluated by Basic Local Alignment Search Tool for nucleotides (BLASTn), where they were analyzed against the homologous nucleotide sequences in the NCBI GenBank. Furthermore, pairwise alignment of similar sequences was performed by using Clustal W in MEGA software (V. 11.0), followed by establishing a maximum-likelihood phylogeny by neighbor-joining method (bootstrap value of 1000 replicates) [92,93].

### 4.4. Preparation of Plant Extract

Fresh leaves of *Piper betle* (about 1 kg) were purchased and washed twice thoroughly with tap water to emancipate dirt, after which they were allowed to dry under direct sunlight. Once dry, they were ground into powder form by mechanical blender, which was stored in air-tight bottles until further use. The fine powder (50 g) was macerated in 1 L each of methanol, ethanol, and chloroform for a period of seven days at room temperature with occasional stirring. After seven days, Whatman filter paper (No. 1) was used to filter the solutions, which were then proceeded under low pressure (50 °C) by using a rotary evaporator (Heidolph) and vacuum pump (V700, BUCHI), which was attached to a chiller (ZGSI). The extracts in crude form were obtained and allowed to dry at room temperature, followed by weighing and storing them in sterile vials in the refrigerator. For achieving a desired concentration (100 mg mL^−1^), the extracts were weighed and allowed to dissolve into their respective solvents [66].

### 4.5. Phytochemical Screening of P. betle Extract

Prepared *P. betle* extracts were evaluated for the presence of various phytochemical compounds (steroids, alkaloids, phenolic compounds, saponins, flavonoids, tannins, terpenoids, carbohydrates, and glycosides) by standard tests [85,94,95].

### 4.6. Thin Layer Chromatography (TLC) Analysis of P. betle Extract

TLC analysis was performed on aluminum sheets (20 × 20 cm; 0.2 mm thickness) pre-coated with silica gel (60 F254, Merck). The plates were prepared by marking one end of the plate with a pencil at the 1 cm mark. The extract sample (2 µL) was added as a spot on the 1 cm mark. The plates were developed in solvent mixture of butanol, methanol, and water (1:1:1 *v/v*) in a glass chamber. They were kept in the tanks until the developing solvent reached the top line, after which they were quickly taken out and allowed to dry. The spots observed for each separate compound on the plate were encircled, and the retardation factor (Rf) value was calculated for each compound according to the following formula [96]:

$$R_{f}\;\mathrm{value}=\frac{Distance\;travelled\;by\;solute\;from\;the\;sample\;spot}{Distance\;travelled\;by\;the\;solvent\;mixture}$$

### 4.7. Fourier Transform Infrared Spectroscopy (FTIR) Analysis of P. betle Leaf Extract

The FTIR Spectrometer (Alpha P; Bruker, Germany) was used, which was interlinked to desktop operated Windows system to connect with OPUS Data Collection software (Version 7.5; Bruker, Germany). Prior to the analysis, the attenuated total reflectance (ATR) plate was cleaned thoroughly by using ethanol (70%), after which it was dried with a soft tissue. For loading the sample, one drop of *P. betle* extract was placed onto the crystal of the ATR plate, while keeping the anvil arm in upright position by active rotation. The FTIR spectra were obtained with the addition of 100 scans in the range of 4000–400 cm^−1^ at a resolution of 4 cm^−1^ [97].

### 4.8. Gas Chromatography–Mass Spectrometry (GC-MS) Analysis of P. betle Leaf Extract

The GC-MS analysis was performed using Agilent GCMS 5975 C gas chromatograph (GC 7890 A) and mass spectrometer (MS 5975 C), equipped with a capillary column (HP-5MS) (30 m × 250 µm × 0.25 µm). Helium was the inert gas acting as a carrier in the column, with a flow rate of 0.8 mL/min (pressure 5.8112 psi, average velocity 32.756 cm/s, holdup time 1.526 min). The injection volume was 1 µL of sample inserted manually. The temperature of the oven was programmed from 5 °C/min to 70 °C/min and 10 °C/min to 300 °C at 240 °C with a hold for 4 min. The MS ionic source and interface were regulated at 240 and 200 °C, respectively. The mass scan range of low and high mass was 30–700 m/z, with a solvent delay of 4 min. Total run time of the analysis was 29 min. Compounds analyzed were verified by comparison of MS spectra with NIST MS Search Library, USA [98].

### 4.9. Minimum Inhibitory Concentration (MIC)

The MIC for the selected bacterial isolate was performed using double macrodilution assay according to the NCCLS broth dilution method [99]. *P. betle* extract was dissolved in Mueller Hinton Agar (MHA) medium, after which the extract was diluted to obtain various concentrations (50, 25, 12.5, 6.3, 3.2, 1.6, 0.8, 0.4, and 0.2 mg mL^−1^). The 0.5 McFarland standard was used to standardize bacterial cultures under sterile conditions. Piperacillin/Tazobactum was used as control. Overnight incubation of test and control tubes was done at 37 °C. The MIC values were observed after successful incubation.

### 4.10. Screening of Antibacterial Activity

For the antibacterial activity of *P. betle* leaf extracts, concentrated extracts were prepared according to variable concentrations chosen for the study (100, 75, 50, 25, and 10 mg mL^−1^) [100]. Sterile Luria Bertani (LB) agar plates were prepared and streaked by pure bacterial cultures. Wells in the plates were made by punching the agar under sterile conditions. Dimethyl sulfoxide (DMSO) and Piperacillin/Tazobactum (100 mg mL^−1^) served as controls in the experiment. The extracts were added in the wells (20 µL), after which they were incubated overnight at 37 °C. Clear zones of inhibition, if any, were discerned and measured for their size in mm [76].

### 4.11. Isolation and Purification of Bioactive Compound from P. betle Extract

The isolation of the bioactive compound of *P. betle* chloroform extract was done according to the method of Khan et al. [101]. Glass column was washed using water (distilled) and allowed to dry. Silica gel served as the stationary phase, and the extract was allowed to run through the column. The isolated fractions were then evaluated for the antibacterial efficacy of the bioactive compound against *B. gaemokensis*.

### 4.12. Time-Kill Assay

Time-kill assay for untreated and untreated *B. gaemokensis* cells was evaluated. Overnight bacterial cultures were inoculated into 100 mL of sterile LB broth. *P. betle* extract (100 mg mL^−1^) was then added, while culture without any extract added, as well as one with an antibiotic, served as the control. Absorbance (OD_595_) was measured at every 1 h interval, after which the data were plotted onto a graph [76].

### 4.13. Assay for Antioxidative Enzymes

For the profiling of antioxidative enzymes, purified bacterial isolates were cultured in Luria Bertani (LB) broth for 24 h. The cultured medium was then supplemented with *P. betle* extract (100 mg mL^−1^) and was again incubated under similar conditions. After a cumulative period of 48 h, the sample was centrifuged for 10 min at 12,000 rpm. The supernatant was discarded, and the pellet was allowed to dissolve in 5 mL of extraction buffer (NaH_2_PO_4_ 50 mm (pH 7.5); PVP 1%; Triton X-100 0.5%; EDTA 1 mm) under cold conditions [102]. The homogenized solution was harvested again, and the supernatant obtained was transferred to a sterile falcon tube and used for analysis of antioxidative enzymes [103,104,105,106,107]. Bacterial cultures without the addition of *P. betle* extract were considered as the control.

### 4.14. Biofilm Experiments

#### 4.14.1. Growth of Biofilms

Sterile LB broth (100 µL) was pipetted in a 96-well microtiter plate, after which bacterial culture (10^7^ mL^−1^) was added to it and then incubated. The following day, bacterial biofilms were observed, and the absorbance was recorded at 600 nm. Biofilm formation was also confirmed by inoculating bacterial culture in sterile LB broth (100 mL) in a flask. After similar incubation conditions, the growth of the biofilm and subsequent absorbance (OD_600_) was observed for confirmation [108].

#### 4.14.2. Inhibition of Established Biofilm

The microtiter plate (96-well) was used to grow bacterial biofilms as described previously. Next, *P. betle* extract was added to the wells, after which the plate was allowed to incubate for 24 h at 37 °C. In the next step, bacterial biofilms were stained with crystal violet (1%), after which water (distilled) was used to wash off excessive stain. Methanol (150 µL) was added to each well for 15 min, followed by the addition of glacial acetic acid (150 µL) when the former was completely dry. The biomass was then prepared for absorbance at 630 nm. Biofilm inhibition (%) was calculated [108]:

[(OD(control) − OD(test)/OD(control)] × 100

#### 4.14.3. Demolishing the Established Biofilm

Bacterial biofilm was allowed to grow as described earlier. The next day, planktonic cells were discarded gently by washing the wells thrice with distilled water. Then, *P. betle* extract (100 µL) was added to the wells and placed for further incubation at 37 °C. Biofilms formed by adherent cells were stained using crystal violet method to assess the disruption of the established biofilm. Biofilm reduction (%) was calculated using the formula [108]:

[(OD(control) − OD(test)/OD(control)] × 100

### 4.15. Protein Estimation

#### 4.15.1. Bradford Assay

Unknown protein samples were prepared using bacterial cultures and *P. betle* extracts (100 mg mL^−1^), whereas cultures without extracts were regarded as the control for the experiment. Readings were taken at 595 nm against LB broth as a blank, with the protein concentration being quantified by a standard curve (1–30 µg) of Bovine Serum Albumin (1 mg mL^−1^) in the presence of the Bradford reagent [109].

#### 4.15.2. Protein Estimation by SDS-PAGE

The effect of *P. betle* extract on bacterial proteins was assessed by SDS-PAGE. Treated and untreated *B. gaemokensis* cells were incubated at suitable conditions. After subsequent incubation, samples were harvested for 10 min at 15,000 rpm. Pellets were homogenized in 20 mM Tris base (pH 7.0). Samples were heated (95 °C for 5 min) to ensure denaturation. Prepared samples were loaded onto polymerized gels (12% resolving, 5% stacking), and power was applied to allow for the bands to migrate to the bottom of the gel. Following electrophoresis, the gel was subsequently stained by staining solution, after which the gel was destained overnight [110].

### 4.16. Scanning Electron Microscopy (SEM)

The growth of untreated and treated *B. gaemokensis* cells with *P. betle* extract was observed through scanning electron microscopy (SEM). Sterile bacterial culture was incubated at suitable conditions, prior to which it was treated with extract. The following day, the cells were centrifuged for 15 min at 10,000 rpm. The pellet was washed with autoclaved distilled water, after which cells were then fixed and dehydrated using glutaraldehyde and ethanol solutions, respectively. The sample was then loaded onto metal stubs, and the images were taken using Nova NanoSEM 450 scanning electron microscope at 10,000× magnification with 5.00 kV as the accelerating voltage [111].

### 4.17. In silico Studies

#### 4.17.1. Network Pharmacology Studies

##### Retrieval of Bioactive Compounds of *P. betle* Extract

Phytochemical compounds previously elucidated by GC-MS were screened for bioactive compounds through extensive literature search at the TCMSP database (http://tcmspw.com/tcmsp.php accessed on 28 August 2022). These phytocompounds were then validated for their bioactivity, and their related genes and protein targets were then accessed and standardized through UniProtKB and the Kyoto Encyclopedia of Genes and Genome (KEGG), respectively [112].

##### Development of PPI Network

The STRING database (https://string-db.org/ accessed on 28 August 2022) and its plugin Cytoscape was utilized to develop the protein–protein interaction (PPI) network between caries and bioactive compounds of *P. betle*. The confidence score of the network was set at 0.4, and the results were filtered for *Homo sapiens* [113].

##### GO and KEGG Pathway Enrichment Analysis

The Gene Ontology (GO) analysis and Kyoto Encyclopedia of Genes and Genomes (KEGG) enrichment analysis were performed by Cytoscape and its plug-in ClueGO. Three primary terms of cellular components (CC), biological processes (BP), and molecular functions (MF) were used to define key genes and their enrichment. Enrichment charts were made on bioinformatic platform (http://www.bioinformatics.com.cn/ accessed on 28 August 2022) [114].

#### 4.17.2. Molecular Docking Analysis

##### Selection and Preparation of Targets and Ligands

Target proteins were obtained by performing an extensive literature survey to highlight those proteins actively involved in caries progression and pathogenesis. Phytochemical compounds previously confirmed by network pharmacology analysis were also verified again by literature search. 3D protein structures were retrieved from the protein data bank (PDB) (www.rcsb.org accessed on 28 August 2022) and were visualized by BIOVIA Discovery Studio tool. Ligand structures were taken from PubChem, after which sdf format was converted into .pdb format by Openbabel software.

##### Molecular Docking Analysis

For molecular docking, the complete protein molecule in .pdb format was converted to pdbqt format, representing the charged entity. AutoDockTools (Version 1.5.6) was used to add hydrogen and charges that were removed during X-ray crystallography. After localizing the amino acids on the respective active sites, the grid box was defined. All the information including the grid box axis, x, y, z centers, ntps, and exhaustiveness were saved in a text file in the working directory and retrieved when needed [115]. The total number of runs were 9, giving the output in 9 different poses. The best pose was selected based on the highest binding affinity.

### 4.18. Statistical Analysis

All control (positive and negative) and experiment set-ups were performed in triplicate under similar experimental conditions. The mean value, standard error, and deviation were evaluated using SPSS (V. 27).

## 5. Conclusions

To the best of our knowledge, this is the first study to report *B. gaemokensis* to be isolated from dental caries. It is widely known that *P. betle* leaf and its extracts serve great biological significance as a natural preservative and antioxidant, antimicrobial, anticancer, and flavoring agent. This study suggests the antibacterial role of *P. betle* chloroform extract against *B. gaemokensis* for the first time, while also revealing its anti-biofilm role. Moreover, the investigation of effective and non-toxic novel bioactive compounds derived from natural sources is vital. This could benefit the development of herbal drugs and formulations to treat human diseases greatly, but great emphasis should be laid upon the isolation and characterization of single active phytocompounds and elucidating their mechanism of action.

## Figures and Tables

**Table 1:** Abbreviations and their full forms.

Abbreviations	Full Form
TLC	Thin layer chromatography
FTIR	Fourier transform infrared
GC-MS	Gas chromatography–mass spectrophotometry
MIC	Minimum inhibitory concentration
SOD	Superoxide dismutase
APOX	Ascorbate peroxidase
POX	Peroxidase
GR	Glutathione reductase
sSDS-PAGE	Sodium dodecyl polyacrylamide gel electrophoresis
SEM	Scanning electron microscopy
GO	Gene Ontology
KEGG	Kyoto Encyclopedia of Genes and Genome
EPS	Extracellular polymeric substances
Rf	Retention factor
PPI	Protein–protein interaction
FabI	Enoyl-acyl carrier protein reductase
MurB	Udp-n-acetylenolpyruvoylglucosamine reductase
FtsZ	Filamenting temperature-sensitive mutant Z
ECM	Extracellular matrix
ROS	Reactive oxygen species
LB	Luria Bertani
CTAB	Cetyl Trimethyl Ammonium Bromide
UV	Ultraviolet
gDNA	Gemonic DNA
DMSO	Dimethyl sulfoxide
OD_595_	Optical density at 595 nm
TCMSP	Traditional Chinese Medicine database and analysis platform

**Table 2:** Details of samples collected for this study.

Samples	Frequency
Total cariogenic samples	2500
Total control	200
Positive cultures	1900
Negative cultures among 2500	600
Highest frequency of cariogenic bacterial flora	165

**Table 3:** Details about the bacterial species isolated from dental caries and control samples.

Category	Bacterial Strains	Morphological Characterization	Molecular Characterization	Accession Number	Biofilm Formation		Frequency Gender (n = 1900)		
					**Sucrose (-)**	**Sucrose (+)**		**Male**	**Female**
Caries-associated bacterial strains Control	VJ-1	Gram positive cocci	*Staphylococcus haemolyticus*	MW067134	+++	+	1199 (63.10%)	721 (61.03%)	478 (39.87%)
	VJ-2	Gram positive rods	*Bacillus flexus*	MW067135	+++	++	1104 (58.01%)	494 (44.75%)	610 (55.25%)
	VJ-3	Gram positive rods	*Bacillus subtilis*-strain 1	MW067136	+	+	1200 (63.15%)	649 (54.08%)	551 (45.29%)
	VJ-4	Gram positive rods	*Bacillus subtilis*-strain 2	MW067137	++	+	1000 (52.63%)	482 (48.20%)	518 (51.80%)
	VJ-5	Gram positive rods	*Bacillus gaemokensis*	MW067143	+++++	+++	1511 (79.52%)	809 (53.54%)	702 (46.46%)
	VJ-6	Gram positive rods	*Bacillus subtilis*-strain 3	MW067139	+++	+	950 (50%)	458 (48.21%)	492 (51.79%)
	VJ-7	Gram positive rods	*Bacillus pumilis*	MW067140	+	+	1428 (75.15%)	661 (46.29%)	767 (46.29%)
	VJ-8	Gram negative rods	*Pseudomonas stutzeri*	MW067141	+	+	998 (52.52%)	521 (52.20%)	477 (47.80%)
	VJ-9	Gram positive rods	*Bacillus cereus*-strain 1	MW067142	++	+	1298 (68.31%)	655 (50.46%)	643 (49.54%)
	VJ-10	Gram positive rods	*Bacillus cereus*-strain 2	MW067138	+	++	1311 (69%)	611 (46.61%)	700 (53.39%)
	VJ-11	Gram positive cocci	*Staphylococcus aureus*	MW067144	+	+	1644 (86.52%)	865 (52.62%)	779 (47.38%)
	VJ-12	Gram positive cocci	*Streptococcus mutans*	NO171456.1	+++	+	1877 (98.78%)	998 (53.17%)	879 (46.83%)
	VJ-13	Gram positive cocci	*Streptococcus sobrinus*	NO171474.1	+	+	1795 (94.47%)	934 (52.03%)	861 (47.97%)
	VJ-14	Gram positive rods	*Lactobacillus salivarius*	NO171459.1	++	+	138 (7.263%)	61 (44.20%)	77 (55.80%)
	VJ-15	Gram positive rods	*Lactobacillus rhamnosus*	NO171462.1	++	+	114 (6%)	60 (52.63%)	54 (47.37%)
	SS-VJ-1	Gram positive cocci	*Staphylococcus aureus*	MW067144	+	+	200 (100%)	90 (45%)	110 (55%)
	SS-VJ-2	Gram positive rods	*Lactobacillus* strain	NO171459.1	++	+	115 (57.5%)	65 (56.52%)	50 (56.52%)

**Table 4:** Phytochemical screening of *P. betle* leaf extract.

Phytocompounds	Test	Chloroform Extract
Alkaloids	Mayer’s test	+
	Wagner’s test	+
Flavonoids	Ferric chloride test	+
	Alkaline reagent test	+
Phenolic compounds	Ferric chloride test	+
Steroids	Liebermann’s test	+
Tannins	Ferric chloride test	+
Glycosides	Liebermann’s test	-
Saponins	Foam test	-
Carbohydrates	Molisch’s test	-
Proteins	Biuret test	-
Amino acids	Ninhydrin test	-
Oils	Spot test	-

**Table 5:** GC-MS analysis of *P. betle* chloroform extract.

Compound	RT	MF	MW
Bicyclo [3.1.1] heptane,6,6-dimethyl-2-methylene-,(1S)-	4.629	C_10_H_16_	136
Benzene,1,2(methylenedioxy)-4-propenyl-,(E)-	9.716	C_10_H_10_O_2_	162
1,3-Benzodioxole,5-(1-propenyl)-	9.957	C_10_H_10_O_2_	162
alfa-Copaene	10.349	C_15_H_24_	204
Eugenol	10.688	C_10_H_12_O_2_	164
Caryophyllene	10.929	C_15_H_24_	204
β- Ylangene	11.607	C_15_H_24_	204
Humulene	11.381	C_15_H_24_	204
γ- Muurolene	11.637	C_15_H_24_	204
Napthalene,1,2,3,5,6,7,8,8a-octahydro-1,8a-dimethyl-7-(1-methylethenyl)-[1R-(1α,3aβ,4α,7β)]-	11.818	C_15_H_24_	204
Azulene,1,2,3a,4,5,6,7- octahydro-1,4-dimethyl-7-(1-methylethenyl)-[1R-(1α,3aβ,4α,7β)]-	11.924	C_15_H_24_	204
Naphthalene,1,2,3,5,6,8a-hexahydro-4,7,-dimethyl-1-(1-methylethyl)-,1S- cis)-	12.210	C_15_H_24_	204
β- Guaiene	12.670	C_15_H_24_	204
α- acorenol	13.951	C_15_H_26_O	222
Phytol	18.329	C_20_H_40_O	296
Phenol,2,2-methylenebis [6-(1,1-dimethylethyl) -4- methyl-	21.109	C_23_H_32_O_2_	340
Diisooctyl phthalate	21.946	C_24_H_38_O_4_	390
Spirost-8-en-11-one,3-hydroxy-,(3β,5α,14β,20β,22β,25R)	22.955	C_27_H_40_O_4_	428
2,2,4-trimethyle-3-(3,8,12,16-tetramethyl-heptadeca-3,7,11,15-tetraenyl)-cyclohexanol	23.905	C_30_H_52_O	428
Pregnane -3,20β-diol,14α,18α-[4-methyl-3-oxo-(1-oxa-4-azabutane-1,4-diyl]-,diace	25.691	C_28_H_43_NO_6_	489

**Table 6:** Growth of *B. gaemokensis* cells at different MIC values.

MIC (mg mL^−1^)	Growth of Bacteria
0	+++
25	+++
50	++
100	-
200	-
250	-
300	-

**Table 7:** Antibacterial activity of *P. betle* extracts against *B. gaemokensis*.

Plant Extract	Concentration	Zone of Inhibition
Chloroform	100 mg mL^−1^	7 mm
Methanol	100 mg mL^−1^	-
Ethanol	100 mg mL^−1^	2 mm
**Negative control**	**Concentration**	**Zone of Inhibition**
DMSO	100 mg mL^−1^	-
**Positive control**	**Concentration**	**Zone of Inhibition**
Piperacillin/Tazobactum	100 mg mL^−1^	6 mm

**Table 8:** Separated column fractions and their antibacterial activity against *B. gaemokensis*.

Column Fractions	Zone of Inhibition (mm)
F1	3
F2	4.1
F3	5.7
F4	12
F5	4
F6	4.5

**Table 9:** Node degree of associated proteins in PPI network obtained through STRING database.

Targets	Node Degree	Targets	Node Degree
ESR1	13	MAOB	2
SLC6A2	4	ADRA1D	4
CHRM3	3	CHRNA2	2
IL6	12	ADRA2C	4
SLC6A3	5	NCOA2	10
ADRA2A	6	RXRA	6
ADRB2	7	IL6R	3
CHRM1	7	NCOA3	9
ADRA1B	5	SRC	18
CHRM2	2	AR	11
PGR	12	SP1	10
MAOA	3	BRCA1	8
PTGS1	4	EP300	10
PTGS2	8	IL6ST	3
ADRB1	4	ALOX5	6

**Table 10:** Binding energies of phytochemical compounds with selected target sites.

Ligands	FabI (kcal/mol)	MurB (kcal/mol)	FtsZ (kcal/mol)
Bicyclo [3.1.1] heptane,6,6-dimethyl-2-methylene-,(1S)-	−6.5	−5.5	−6.0
Benzene,1,2(methylenedioxy)-4-propenyl-,(E)-	−6.8	−6.2	−7.4
1,3-Benzodioxole,5-(1-propenyl)-	−6.5	−6.7	−7.3
Alpha-Copaene	−8.3	−6.8	−5.9
Eugenol	−6.1	−6.3	−6.8
Caryophyllene	−8.8	−6.4	−6.5
β-Ylangene	−8.1	−6.7	−7.6
Humulene	−6.1	−6.2	−6.6
γ- Muurolene	−8.5	−6.0	−7.9
Napthalene,1,2,3,5,6,7,8,8a-octahydro-1,8a-dimethyl-7-(1-methylethenyl)-[1R-(1α,3aβ,4α,7β)]-	−8.4	−6.4	−8.0
Phytol	−3.8	−4.7	−4.2
α- acorenol	–	–	–
β- Guaiene	–	–	–
Diisooctyl phthalate	−7.9	−9.3	−9.3
Spirost-8-en-11-one,3-hydroxy(3β,5α,14β,20β,22β,25R)	−12	−17.1	−14.9
2,2,4-trimethyle-3-(3,8,12,16-tetramethyl-heptadeca-3,7,11,15-tetraenyl)-cyclohexanol	−7.1	−6.0	−6.0
Naphthalene,1,2,3,5,6,8a-hexahydro-4,7-dimethyl-1-(1-methylethyl)-,1S- cis)-	−7.2	−8.1	−6.3
Azulene,1,2,3a,4,5,6,7- octahydro-1,4-dimethyl-7-(1-methylethenyl)-[1R-(1α,3aβ,4α,7β)]-	–	–	–
Phenol,2,2-methylenebis [6-(1,1-dimethylethyl) -4- methyl-	–	–	–
Pregnane -3,20β-diol,14α,18α-[4-methyl-3-oxo-(1-oxa-4-azabutane-1,4-diyl]-,diace	–	–	–

## Data Availability

Not applicable.
